# Intersectionality analysis of young people’s experiences and perceptions of discrimination in primary health centers in Ebonyi State, Southeast Nigeria

**DOI:** 10.1186/s12939-024-02192-6

**Published:** 2024-05-17

**Authors:** Chibuike Innocent Agu, Ozioma Nwankpa, Chinazom N. Ekwueme, Ifunanya Clara Agu, Chinyere Ojiugo Mbachu, Nkoli Ezumah, Obinna Onwujekwe

**Affiliations:** 1 Health Policy Research Group, University of Nigeria Nsukka, Enugu, Enugu State Nigeria; 2 Department of Community Medicine, Alex-Ekwueme Federal University, Ndufu-Alike, Ebonyi State Nigeria; 3 Department of Community Medicine, College of Medicine, University of Nigeria Nsukka, Enugu, Enugu State Nigeria; 4 Department of Health Administration and Management, College of Medicine, University of Nigeria Nsukka, Enugu, Enugu State Nigeria

**Keywords:** Adolescents and young people, SRH, Primary health centers, Discrimination, Experiences, Perception, Intersectionality

## Abstract

**Background:**

Young people (aged 10 to 24 years) in sub-Saharan Africa bear a huge and disproportionate burden of poor sexual and reproductive health (SRH) outcomes due to inequalities and discrimination in accessing sexual and reproductive health services (SRHS). This study assessed the experiences and perceptions of discrimination among young people seeking SRH services in Primary Health Centers (PHCs) using an intersectionality lens.

**Methods:**

A cross-sectional mixed-methods study was undertaken in six local government areas (LGAs) in Ebonyi State, southeast Nigeria. The LGAs comprise both urban and rural locations. The study population for the quantitative survey consisted of 1025 randomly selected young boys and girls aged 15–24 years. Eleven focus group discussions (FGDs) were conducted with the young people. Descriptive and inferential analyses were performed for quantitative data, while thematic analysis was performed for the qualitative data, using NVivo.

**Results:**

A total of 16.68% participants in the survey reported that young girls/women were treated badly/unfairly compared to young boys/men when seeking SRH services in PHCs; 15.22% reported that young clients get treated badly/unfairly from adults; and 12.49% reported that young clients with poor economic status were treated unfairly. Respondents also reported that young clients with disability (12.12%), and those who are poorly educated or uneducated (10.63%) are treated badly by healthcare providers when they access SRH services. Young people in urban areas were about 7 times more likely to believe that girls/young women are treated badly than boys/young men when seeking SRH services in PHCs compared to those who live in rural areas (*p* < 0.001). Among the young girls/women, residing in urban areas, being poor and in school increased the likelihood of getting treated badly/unfairly when receiving SRH services by 4 times (*p* < 0.001). The qualitative results revealed that health workers were generally harsh to young people seeking SRH services and the level of harshness or unfriendliness of the health workers varied depending on the young person’s social identity.

**Conclusion:**

There are varieties of intersecting factors that contribute to the discrimination of young clients in PHCs. This underscores the urgent need to prioritize intersectional perspectives in the design and implementation of interventions that will improve access and use of SRH services by young people.

## Background

In sub-Saharan Africa (SSA), young people (aged 10 to 24) continue to bear a huge and disproportionate burden of poor sexual and reproductive health (SRH) outcomes [1, 2]. The rates of teenage pregnancy, unsafe abortion, child marriage, sexually transmitted infections, and unmet need for contraception among young people are substantially higher in SSA compared to other parts of the world [3–5]. In addition, pregnancy and childbirth in young people are associated with adverse maternal and neonatal outcomes, such as elevated risks for low birth weight, preterm delivery, severe neonatal conditions, early neonatal deaths, maternal mortality, and morbidity [6–8].

To address the unique needs of young people, the World Health Organization has highlighted the need for youth-friendly health services. These are health services that are tailored to young people’s needs and can contribute to improved utilization of SRH services, and overall better health outcomes [9]. Youth-friendly SRH services include SRH education, HIV counseling provision of contraceptives, treatment of sexually transmitted infections (STIs), management of unsafe abortion, pregnancy, and other SRH services [9].

Nigeria is regarded as a high-burden country for adolescent and youth sexual and reproductive health issues [10]. The country accounts for the second-highest number of maternal deaths in the world, and adolescents and young people are significant contributors to this burden [11]. Nigeria also has one of the highest burdens of the Human Immunodeficiency Virus (HIV) globally and young people aged 15–24 years account for about a third of new HIV infections [12]. Reports show that 36 per 1000 women of reproductive age in Nigeria have had an abortion, and this is highest among young women and girls [12–14]. The rate of abortion in Nigeria is higher than the regional average of 28 per 1000 in sub-Saharan Africa [12–14].

Young people’s access to SRH services is still relatively poor in Nigeria as in several other SSA countries. This is often because, even when SRH services are available, young people face several other barriers to accessing these services [15–17]. These barriers include provider disapproval, concerns about violations of confidentiality, past experiences of embarrassment, restrictive gender norms, societal shaming, and unfriendly attitudes of healthcare providers [17, 18]. Sometimes, service providers struggle with the legal and moral responsibilities of providing services to adolescents and young people [18, 19]. Also, they are often discriminated against based on certain identity markers or factors, such as gender, social class, economic status, sexual orientation, religion, age, and mental or physical disability, among others.

Hence, in response to recommendations by WHO, Nigeria has developed policies for youth-friendly services such as the National Guideline for Integration of Adolescent and Youth-Friendly Services into Primary Healthcare Facilities, and the National Policy on the Health and Development of Adolescents and Young People in Nigeria [20, 21].

However, there are still glaring inequalities in access to and utilization of SRH services. This underscores the need to employ intersectionality lens to understand complex and interrelated factors that affect young people’s access to SRH services [22].

Although a good number of studies have examined the barriers to adolescents and young people’s access to SRH services in Nigeria, none of these studies applies the intersectionality framework [17, 18, 23, 24]. Intersectionality analysis offers a useful starting point for analyzing the overlapping identities (interacting factors) and how these intersections contribute to unique experiences of discrimination and privilege [25]. It considers the social and historical context of the group as well as the fact that discrimination has evolved and tends to be more subtle, multi-layered, systemic, environmental, and institutionalized [26].

This paper provides new knowledge on how different sets of identities impact young people’s access to youth-friendly sexual and reproductive health services in PHCs in Ebonyi State, Nigeria. Thus, it provides new evidence on social identities and factors that policymakers and program planners may find useful for transformative policy solutions to achieve the targets of Sustainable Development Goal 3 regarding access to SRH services for young people.

### Conceptual framework

Intersectionality is both a theoretical and methodological ‘lens’ that offers a fresh perspective on the complex interactions of social identity markers/factors in ways to produce systems of oppression, discrimination, or privileges. It recognizes the complicated nature of how people experience discrimination, acknowledging that each person’s experience may be distinct [27, 28]. Intersectionality is increasingly being used within public health research in lower and middle-income countries [29].

Originally coined by Kimberlé Crenshaw in the USA in response to the exclusion of black women from feminist theory, the intersectionality framework is a useful tool for analysis, advocacy, and policy development that addresses multiple discriminations, and helps in understanding how different sets of identities influence access to rights and opportunities [25].

**Fig. 1:**
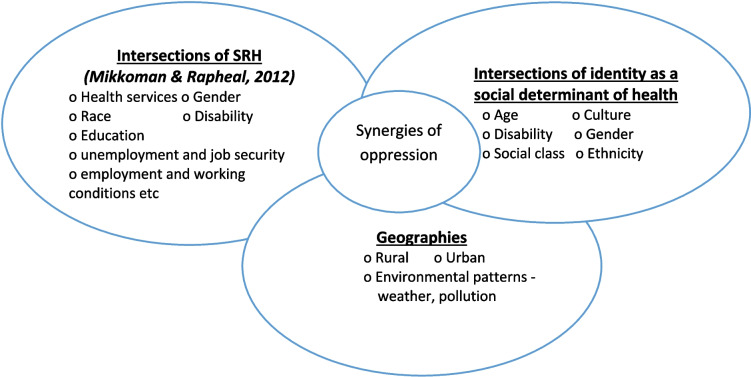
An intersectionality framework, adapted from the synergies of oppression [32]

As a theoretical paradigm, it helps to explore the convergence of different social identifiers, types of exclusion, and marginalization within a population or an individual [30]. Overlapping systems of oppression or discrimination, such as gender, race, economic status, disability, etc., shape the social identities of people, reinforcing existing power structures and privileges, as well as producing synergies of oppression [28] (Fig. 1). For instance, gender inequality is often mutually reinforced by other forms of inequality, including racism, homophobia, and economic elitism [31].

## Methods

### Study design and study area

This cross-sectional mixed methods study was undertaken in six out of the thirteen local government areas (LGAs) of Ebonyi State, Nigeria. The quantitative approach was employed to enable us quantify young people’s experiences and perceptions of discrimination, and to identify the interactions of factors associated with these. The qualitative approach was used to explore the experience of discrimination among these young people.

Three rural and two urban LGAs were purposively selected for this study, including, Abakaliki, and Izzi LGAs in Ebonyi North senatorial district, Ezza South, and Ikwo LGA in Ebonyi Central senatorial district, and Ohaozara, and Afikpo-south in Ebonyi south senatorial district. With an annual growth rate of 2.8%, Ebonyi State has an estimated total population of 4,339,136, out of which 355,000 are young people aged 15–24 years [33, 34].

### Study population, and sampling techniques

The study population for the quantitative research consisted of young boys and girls within 15–24 years regardless of schooling or marital status. The six LGAs with the poorest sexual and reproductive health (SRH) outcomes among young people were purposively selected from the 3 senatorial zones in the state. The LGAs were prioritized by the State government and partners for scaling up SRH interventions. In each LGA, a community was selected based on the recommendation of an established primary healthcare facility that provides youth-friendly SRH services.

The respondents were selected from six communities using a modified cluster sampling technique. Our definition of a cluster was an autonomous community governed by a traditional ruler. The nearest public structure (such as a school, church, community hall, etc.) from the entrance of the community was used as the starting point from which households were consecutively selected. Eligible participants from selected households were required to give consent before participating in the study.

For the qualitative aspect of the study, young people aged 15–24 were purposively drawn from the survey participants. Young people who had utilized the youth-friendly SRH services from healthcare facilities in the communities were invited to participate in the focus group discussions (FGDs). The participants included young people in or out of school, and those who are married or unmarried.

### Sample size calculation

A sample size of 606 households was determined using the guidelines outlined in the demographic and health survey (DHS) listing manual using the formula [35]:

$$n={Deft}^2\times\frac{\frac1p-1}{\alpha^2}$$

Where *n* is the sample size to be calculated, *Deft* is the design effect (i.e., the ratio between the standard error using our sample design and the standard error that would result had we used a simple random sampling), $p$ is the estimated proportion of the attribute present in the population, and α is the desired relative standard error. The design effect was set at 1.6, which is a lower bound of what DHS indicators produced. We set $p$ at 0.5, and the desired relative standard error $\alpha$ at 0.065.

Plugging these values into the above formula gives *n* ≈ 606. To arrive at the 606 households, 101 households were drawn on a cluster basis from each of the six purposively selected LGAs with PHCs that provide youth-friendly sexual reproductive services serving as clusters. To estimate the sample size for young people, we maintained the same values for the proportion 𝑝 and the design effect *Deft* but set the standard error 𝛼 at 0.0501. Therefore, the estimated average *n* ≈ 1,020. However, a total, of 1,025 young people (aged 15 to 24 years) were drawn from the selected 606 households.

### Data collection

The quantitative data collection instrument was adapted from an annual publication on gender and evaluation [36] and was pilot-tested in Enugu state, southeast Nigeria.

The qualitative data were collected from December 2022 to March 2023 using a pre-tested focus group discussion (FGD) guide. A total of eleven FGDs were held with young people aged 15–24 years with an average of 6 participants per FGD. They were disaggregated by sex (male/ female) and age (15–18 and 19–24). The FGD sessions were conducted by experienced social scientists who were briefed on the objectives of the study and the purpose of the FGDs. All the interviews were conducted in the English language because the young people could understand the language. However, young people who wished to express themselves in local dialect were allowed to do so. Participants were informed of the objectives of the study and their roles and rights in it. Written consent was obtained from each participant before the interviews began. The discussions were held in venues that were convenient for participants while ensuring confidentiality.

### Data analysis

#### Quantitative data analysis

Univariate and multiple logistic regression analyses were performed using STATA statistical software. Means with standard deviations, proportions, and percentages were used for the univariate analyses.

The multiple logistic regression analysis model (with and without interaction) was used in this study. The multiple logistic regression analysis model allowed us to extend the analysis by isolating predictors of young people’s perception of discrimination based on gender, age, education, economic and disability status while considering variations in individual, interpersonal, and social-level factors under a regression framework. The fitting of a model that includes interaction terms was necessary to estimate the effects of intersectionality on their perceptions of discrimination against young people seeking SRH care. Through this, we demonstrated the main effect of the independent variables (age, sex, area of residence, schooling status, marital status, and working status) on the one hand and, on the other hand, the interactions between the male and female gender and the different combinations of these variables.

The outcomes of interest included perceptions of the occurrence of discrimination in PHCs. Data were collected on the respondents’ experiences and perceptions of the occurrence of discrimination based on certain characteristics, such as gender, age, economic status, disability status, and educational status, by stating “yes” or “no” to some statements. These statements include i.) Girls/young women get treated differently (badly/ unfairly) from boys/young men when they seek sexual or reproductive health services; ii.)Young people get treated differently (badly/ unfairly) from adults when they seek sexual or reproductive health services; iii.)Young people get treated differently (badly/ unfairly) when they seek sexual or reproductive health services if they are poor; iv.)Young people get treated differently (badly/ unfairly) when they seek sexual and reproductive health services if they are disabled; and v.)Young people get treated differently (badly/ unfairly) when they seek sexual and reproductive health services if they are illiterate, uneducated, or poorly educated.

The independent variables include (i)individual-level factors - gender, schooling status, age category, and religion (ii)interpersonal-level factors – living with parents/guardian, who is the head of household, father’s highest level of education, and mother’s highest level of education; (iii)social-level factors which included the area of residence (whether urban or rural), and working status. The level of statistical significance was determined by a *p*-value of < 0.05.

#### Qualitative data analysis

The recorded discussions of qualitative interviews were transcribed by the note-takers verbatim following each session. Responses in the local dialect were translated into English language concurrently. The notes were used to assign proper labels to the transcripts and to further enrich the transcripts with nuances and non-verbal cues that were observed during the KIIs and FGDs. Each transcript was read by the interviewer or moderator, and further edited for spelling and punctuation errors.

**Fig. 2:**
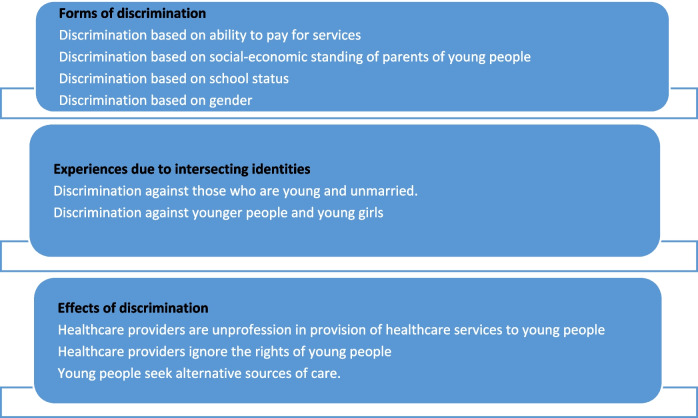
Summary of young people’s experiences of discrimination from the qualitative findings

Thematic analysis of transcripts was performed using a deductive approach. A coding framework was developed based on the objectives of the evaluation. The transcripts were then imported and coded in the NVivo software (release 1.7.1; 1534). The generated word query output was thoroughly read to identify themes as shown in Fig. 2.

## Results

Table 1 shows the socio-demographic characteristics of the respondents. Their mean age was 18.10 (± 2.61). Similar proportions of the respondents lived in the urban (49.76%) and rural (50.24%) areas. There were 454 (44.29%) males and 571 (55.71%) females. Most of the respondents (79.02%), were not working.

**Table 1:** Socio-demographic characteristics of the respondents

Variable (*N* = 1,025)	Frequency (*n*) *N* = 1025	Percentage (%)
Mean age ± std. deviation (in years) 18.10 ± 2.61		
Age category		
15–16years	341	33.27
17–18years	291	28.39
19–20years	205	20.00
21–24years	188	18.34
**Place of residence**		
Rural	515	50.24
Urban	510	49.76
**Sex**		
Female	571	55.71
Male	454	44.29
**Currently in school**		
Yes	647	63.12
No	378	36.88
**Marital status**		
Married	22	2.15
Unmarried	1001	97.66
Refused to say	02	0.20
**The highest level of Education attained**		
None	8	0.78
Primary	605	59.02
Secondary	405	39.51
Tertiary	7	0.68
**Religious affiliation**		
Christian roman catholic	517	50.44
Christian protestant	438	42.73
Other religions (e.g. African tradition, Muslim)	70	6.83
**Is/ has been in an intimate sexual relationship**		
Yes	246	24.00
No	779	76.00
**Living with parents/guardian**		
Yes	977	95.32
No	48	4.68
**Employment status (work for pay)**		
Yes	215	20.98
No	810	79.02

Table 2 shows the participants’ perceptions of whether young people were treated badly or unfairly (that is if they experienced discrimination) based on their gender and other identities. The result shows that 16.68% reported that young girls/women were treated badly/unfairly compared to young boys/men; 15.22% reported that young people were treated badly/unfairly compared to adults; 12.49% reported that young people were treated badly/unfairly if they were poor; 11.12% reported that young people were treated badly/unfairly if they had a disability; and 10.63% reported that young people were treated badly/unfairly if they were illiterate, uneducated or poorly educated.

**Table 2:** Perceptions of differential treatment of young people based on gender and other identities

Variable	Frequency *N* = 1025	Percentage (%)
Forms of Discrimination among young people using SRH services		
Girls/young women get treated differently (badly/ unfairly) from boys/young men when they seek for sexual or reproductive health services	171	16.68
Young people get treated differently (badly/ unfairly) from adults when they seek for sexual or reproductive health services	156	15.22
Young people get treated differently (badly/ unfairly) when they seek sexual or reproductive health services if they are poor	128	12.49
Young people get treated differently (badly/ unfairly) when they seek sexual and reproductive health services if they have a disability	114	11.12
Young people get treated differently (badly/ unfairly) when they seek sexual and reproductive health services if they are illiterate, uneducated, or poorly educated	109	10.63

Table 3 shows the logistic regression of factors associated with girls/young women being treated differently from boys/young men. Young people in urban areas are about 7 times more likely to believe that girls or young women are treated differently from boys or young men compared to those who live in rural areas.

**Table 3:** Logistic regression of factors associated with young people’s perception that “girls/young women are treated badly/unfairly than boys/young men”

Variable (*N* = 1025)	Adjusted odds ratio	*p* value	95% Confidence Interval
**Age**	1.000	0.992	[0.924, 1.082]
**Sex**			
Female	1		
Male	1.040	0.828	[0.731, 1.478]
**Area of residence**			
Rural	1		
Urban	7.290	0.000*(< 0.001)	[4.697, 11.316]
**Currently in School**			
No	1		
Yes	1.389	0.135	[0.903, 2.136]
**Marital Status**			
Married	1		
Single	2.5051	0.240	[0.542, 11.586]
**Currently Working**			
No	1		
Yes	1.651	0.050	[1.000, 2.726]

*Statistical significance (*p*<0.05)

Table 4 shows the intersectionality (interactions) of the factors associated with young people’s perception that “girls/young women are treated differently from boys/young men.” Female respondents who live in rural areas are 8 times less likely to have experienced gender-related discrimination or perceive that young women or girls are being treated differently from boys or young men compared to female respondents who live in urban areas.

**Table 4:** Intersectionality (interactions) of the factors associated with young girls/women’s perception of gender-based discrimination of young women

Variable (*N* = 571)	Adjusted odds ratio	*p*-value	95% Confidence Interval Lower Upper
Female + Urban	1		
Female + rural	0.125	< 0.001*	[0.068, 0.231]
Female + currently in school	1		
Female + not currently in school	0.812	0.375	[0.514, 1.285]
Female + single	1		
Female + married	0.329	0.286	[0.043, 2.527]
Female + currently working			
Female + not currently working	1.327	0.352	[0.731, 2.407]
Female + urban + schooling	1		
Female + urban + not schooling	0.864	0.590	[0.507, 1.471]
Female + rural + schooling	0.126	< 0.001*	[0.058, 0.277]
Female + urban + not schooling	0.107	< 0.001*	[0.041, 0.278]
Female + rural + schooling + working	1		
Female + rural + schooling + not working	0.091	< 0.001*	[0.024, 0.341]
Female + rural + not schooling + working	0.029	0.002*	[0.003, 0.287]
Female + rural + not schooling + not working	0.106	0.004*	[0.023, 0.481]
Female + urban + schooling + working	1		
Female + urban + schooling + not working	0.632	0.441	[0.197, 2.030]
Female + urban + not schooling + working	0.686	0.587	[0.175, 2.680]
Female + urban + not schooling + not working	0.524	0.302	[0.154, 1.788] 1.788

*Statistical significance (*p*<0.05)

Table 5 shows the logistic regression of factors associated with young people’s perception that “young people get treated differently (badly/ unfairly) when they seek for sexual or reproductive health services if they are poor” Young people in urban areas are about 5 times more likely to get treated differently (badly/ unfairly) when they seek for sexual or reproductive health services if they are poor compared to those who live in rural areas.

**Table 5:** Logistic regression of factors associated with young people’s perception that “young people get treated differently (badly/ unfairly) when they seek sexual or reproductive health services if they are poor”

Variable (*N* = 1025)	Adjusted odds ratio	*p*-value	95% Confidence Interval Lower Upper
**Age**	1.028	0.531	[0.943, 1.121]
**Sex**			
Female	1		
Male	1.134	0.524	[0.770, 1.671]
**Area of residence**			
Rural	1		
Urban	5.179	< 0.001*	[3.230, 8.303]
**Currently in School**			
No	1		
Yes	1.220	0.407	[0.762, 1.952]
**Marital Status**			
Married	1		
Single	0.932	0.917	[0.252, 3.451]
**Currently Working**			
No	1		
Yes	1.005	0.988	[0.566, 1.781]

*Statistical significance (*p*<0.05)

Table 6 shows the intersectionality (interactions) of the factors associated with young girls’ perception that “young people get treated differently (badly/ unfairly) when they seek sexual or reproductive health services if they are poor.” Female respondents who live in rural areas are 5 times less likely to have experienced socio-economic-related discrimination compared to female respondents who live in urban areas. Female respondents who live in rural areas, and who are not in school are approximately 4 times less likely to believe that young people get treated differently (badly/ unfairly) when they seek sexual or reproductive health services if they are poor compared to female respondents who live in urban areas and are in school.

**Table 6:** Intersectionality of the factors associated with young girls/women’s perception that “young people get treated differently (badly/ unfairly) when they seek sexual or reproductive health services if they are poor”

Variable (*N* = 571)	Adjusted odds ratio	*p*-value	95% Confidence Interval
Female + Urban	1		
Female + rural	0.202	< 0.001*	[0. 108, 0.379]
Female + currently in school	1		
Female + not currently in school	0.947	0.198	[0.566, 1.585]
Female + single	1		
Female + married	1.703	0.416	[0.472, 6133]
Female + currently working	1		
Female + not currently working	1.311	0.435	[0.664, 2.591]
Female + urban + schooling	1		
Female + urban + not schooling	0.792	0.457	[0.430, 1.462]
Female + rural + schooling	0.122	< 0.001*	[0 0.047, 0.319]
Female + rural + not schooling	0.276	0.002*	[0.124, 0.617]
Female + rural + schooling + working	1		
Female + rural + schooling + not working	0.558	0.356	[0.162, 1.925]
Female + rural + not schooling + working	0.346	0.188	[0.071, 1.680]
Female + rural + not schooling + not working	0.511	0.314	0.139 to 1.884
Female + urban + schooling + working	0.187	0.164	0.018 to 1.977
Female + urban + schooling + not working	0.062	< 0.001*	0.013 to 0.289
Female + urban + not schooling + working	0.085	0.009*	0.013 to 0.533
Female + urban + not schooling + not working	0.232	0.048*	[0.055, 0.989]

*Statistical significance (*p*<0.05)

### Qualitative findings

Young people were asked to describe their experiences of seeking sexual and reproductive health services from primary healthcare workers. A recurrent theme from the FGDs was that health workers were generally harsh and unfriendly to young people who seek for SRH services. However, the level of harshness or unfriendliness of the health workers varied depending on the young person’s social identity.

#### Young people’s experiences of discrimination

1. Forms of discrimination

The findings from the focus group discussions confirmed that young people experience discrimination while accessing SRH services, on the bases of their gender, schooling status, ability to pay for services, and the socio-economic standing of their parents.

a Discrimination based on the ability to pay for services

Some participants narrated personal experiences of where access to health services was delayed or denied to them or to other young people because they could not pay for the services. They reported that young people who could not afford to pay for services in the healthcare facility are denied medical treatment. Most times when young people are denied access to healthcare, they seek alternative healthcare from patent medicine vendors or self-medication, which potentially results in fatal health consequences. Young girls also reported that some healthcare providers abandon poor young clients and prioritize those who can afford to pay for the services. The following quotes highlight the experiences of some of the participants;

*“Yes. I told them that I don’t have deposit money for my treatments and they told me that they don’t allow credit in their health center. I was angry and asked them if they would refuse to treat an unconscious person and if the person would pay later. But they refused and told me to go and get money before they can treat me.” (FGD with younger girls_YF06, Participant 1)*.

*“One of my friends got pregnant but she didn’t find out early enough. Her parents didn’t know that she was pregnant, she tried her best not to get pregnant but mistakenly she got pregnant. She did something to remove the pregnancy and after that, she started experiencing stomach pain. I followed her to visit the health center but she did not receive the treatment because we did not have enough money to pay for the test she was asked to do. You know that she has already removed the baby from her womb but she lost her life because the bleeding was much just like that this new year. A friend gave her the medication” (FGD with older young girls_OF03, Participant 7).*

b. Discrimination based on socio-economic standing of parents of young people

Some of the participants stated that the financial status of their parents influenced how they were treated by the healthcare workers. They reported that young clients experience denial of health services or can be overlooked by healthcare providers if their parents are of low socioeconomic status in the community. Whereas those whose parents are rich and influential are usually provided with their choice of health services without delay or questioning from healthcare providers. Some resonating quotes are highlighted.

*“When I visited the hospital with my mother, although we are not poor but a middle-class family. When we arrived at the hospital the nurses that were attending to us abandoned us and started attending to a rich family that just come to the hospital. Many people were waiting for her at my back but she took those rich ones inside and treated them first before treating us.” (FGD with older young girls_OF03, Participant 4)*.

*“Yes, the way a poor man’s daughter is treated will not be the same way a rich man’s daughter will be treated.” (FGD with older young boys_OM10, participants 2)*.

*“Sometimes they will overlook you because you don’t have money. You know people nowadays are more interested in money. There is a girl whose family is poor, she had surgery and was having stomach pains, and her old father took her to the health center. But because her family is poor, the health workers overlooked the situation in her pain because she doesn’t have money to pay. They say that their services are not free and that the person should go home if she does not have money” (FGD with younger girls YF06, participants 8)*.

*“To take for instance, if you are pregnant and from a poor family, then you go to a health center and ask them to give you medicine that will make the baby be aborted, they will give you the one that will make your baby grow, but if her parents are rich, they will take her to the hospital and abort the pregnancy” (FGD with younger girls_YF05, participant 5)*.

c. Discrimination based on schooling status

It was also reported that young people who are in school are discriminated against compared to their counterparts who may are not in school. Concerning access, to contraceptive services, young people who visit the healthcare facility and demand contraceptives are questioned and scolded by healthcare providers and reprimanded to focus on their academics.

*“…. Yes, am saying based on experience in school. If you go to the health centre to collect protection for sex, that woman [healthcare provider] there will start by saying is that why you came to school? Why can’t you go and read, …” (FGD with young people_YM02, Participant5)*.

d. Discrimination based on gender

The findings showed that gender-based discrimination of young people was related to the type of SRH service being sought. Two participants highlighted that young girls who present with unwanted pregnancy and post-abortion complications are usually treated poorly by healthcare workers, and very often without recourse to their male partners. In their own words:

*“Sometimes it may be that the person got pregnant from sexual intercourse or sexual assault but they will use it against you, but will not bother about the male partner. That’s some of the things that [female] young teenager considers and won’t have the courage to visit the health workers.” (FGD with older young girls_OF03, Participant 1)*.

*“If post-abortion ladies go there to receive care, those health workers there will neglect them, not minding that a man is responsible for the condition.” (FGD with older young girls_OF03, Participant 5)*.

A female participant recounted an experience when she visited a primary health centre because she was having heavy vaginal bleeding. She described the staff’s spontaneous and humiliating reactions, which involved raining abuses on her and unfounded accusations of attempting an illegal abortion, even without proper history-taking or examination.

*“When I started having my menses new, it used to come in heavy flows. On one occasion, the flow was too much, and the pain too. My friend took me to one of the health centers and immediately I told the nurse that I was bleeding, and she started shouting at me saying that I had an abortion somewhere, and that was why I was bleeding…my boyfriend was with me and nobody talked to him about it.” (FGD with older young girls_OF03, Participant 2)*.

In contrast, a male discussant was of a different opinion that young girls are not discriminated against when they seek contraceptive services. Rather, it is the young boys that are discriminated against. Citing access to condoms as an example, he described his views in the following quote.

*“If you go there to buy something like a condom or drugs, if it is a boy, that person will start by asking you who sent you, and if you don’t answer perfectly, they will tell you to go and call the person that sent you. But if it is ladies that went for it, they will give it to them”* **(** *FGD with older young boys_OM12, Participant 1)*.

2. Experiences due to intersecting identities

Some of the participants described discrimination based on the intersection of certain identities as follows:

- Discrimination against those who are young and unmarried

Both young boys and girls reported that healthcare providers considered the age and marital status of the young client before providing SRH services (including information/counseling, and condoms). Young people were often denied contraceptives including condoms because of their age and marital status. It was also disclosed that in some health facilities, some of the healthcare providers focused more on the provision of SRH services to married or older persons. Married young girls and young boys were treated like every other adult who came to receive SRH services. Young people mentioned that they preferred to seek SRH information and services outside their communities where they are unlikely to be recognized as too young and/or single. Some supporting quotes are shown below:

*“…They don’t give condoms to young people like us, unlike adults or those married (FGD with young people _YM 01, Participant 4)*.

*“You know, in this area, most of the healthcare providers in primary healthcare centers focus more on pregnant women and nursing mothers to teach on sexual and reproductive health than young girls. Except outside this community, that is when you can get such information.” (FGD with young people _YF07, Participant 7)*.

*“Sometimes, it does affect the way they treat young people because they will be looking at you maybe your age and marital status. They will ask you some questions like are you married and your age, but in the case that you are married it will not be a problem because they will provide you with the sexual and reproductive health services that you want” (FGD with young people_OM10, Participant 6)*.

b) Discrimination against younger girls

The age of the young person often intersected gender in their experience of SRH services, particularly contraceptives. Young people mentioned that healthcare providers were not comfortable to provide contraceptives to those who are less than 18 years and subsequently denied them the service when they visited the healthcare facility. The attitudes of the healthcare providers were also influenced by the gender of the young person. The participants explained that younger boys received fewer or no queries from healthcare providers compared to the younger girls.

***“*** *Some of the health workers are not okay with young people of 15 and 16 years coming to ask for condoms. So, they do not give them when they come for it” (FGD with young people_ OM10, Participant 2)*.

*“The difference is that as I am if I go to the healthcare facility, they will ask me some questions, but if it is an older person, they already know what he or she wants to use it for.” (FGD with older young boys_OM12, Participant 6)*.

3) Effects of discrimination

The findings highlight three main effects of the discriminatory attitudes of health workers on young people; (a) feeling of shame; (b) disempowered of their SRH rights; and (c) discouraged to seek SRH services from PHCs.

- Feeling of shame

Some of the young people stated that the harsh and unfriendly treatments that they received from the health workers elicited a feeling of shame and unworthiness from them.

***“…*** *you know there are some nurses that think that they know all, so they will be talking to you as if you are nothing, so I used to feel ashamed about it. But if it is those that are well mannered, if they are talking to you, they will put small respect on it even though you don’t have anything that respect on, it will make you proud” (FGD with older young boys_OM12, Participant 2).*

b. Disempowered to exercise their SRH rights

Young people mentioned that although they know their rights, the healthcare providers often made them feel disempowered to exercise their SRH rights. Young people believe that healthcare providers infringe on the rights to SRH services because of the high level of power they have over their clients.

*“….every individual knows that we all have a right but those healthcare providers who are seated in that facility will always feel their right overwhelms our own; maybe because they are the one rendering services to people so they can just treat you anyhow they want” (FGM YF 07, Participant 5)*.

c. Discouraged to seek SRH services from PHCs

Young people disclosed that the discrimination they face in accessing health services discourages them from utilizing the services provided in primary healthcare centres. This makes them opt for alternative sources of SRH services, like the patent medicine vendors. A supporting quote is highlighted.

*“….like me now, instead of me going to the health centre to open my problem to them, I will rather go to any nearby chemist to buy what I want to buy because the way they do approach their client or patient is not favourable” (FGM YM 02, Participant 4)*.

## Discussions

In this study, intersectionality has been contextualized to highlight the experience of discrimination among different categories of young people seeking SRH services. Findings demonstrate a variety of intersecting factors, such as age, area of residency, gender, schooling status, disability status, and working status, that contribute to young people’s experience of discrimination in SRH services. Multiple identities interconnect in one person to create a whole that is different from each identity or social categorization [31, 37].

Findings from the qualitative data revealed various manifestations of discrimination including rude and unwelcoming attitudes of health workers, and outright denial of SRH services to young people. This is consistent with the findings from several countries in sub-Saharan Africa (SSA) following a review of the barriers to SRH services, access, and utilization among young people in the region from 1994 to 2019 [38]. Indeed, instances of discrimination within the health care system may be more common than previously thought, as noted by a study in the USA [39]. Such discriminatory practices could adversely impact the health outcomes of young people by discouraging and hindering their access to high-quality care, thereby further widening already-existing health disparities.

An unexpected finding from the quantitative data was that participants living in urban areas have higher odds of reporting that girls or young women encounter discrimination compared to their rural counterparts. This is contrary to the results from earlier studies in Ethiopia, Ghana, and Tanzania, which found higher odds of perceived barriers to healthcare access among rural women [40–42]. However, the findings from our study may be due to several factors, such as higher expectations of the treatment they receive among urban young people. There is also the issue of PHCs in urban areas dealing with larger numbers of clients, which often leads to congested clinics, long waiting times, and hurried consultations, thus making healthcare providers appear less attentive, indifferent, or even disrespectful to young people seeking SRH services [43].

The interplay of intersecting identities to produce systems of discrimination among young people who seek SRH services in health facilities is also demonstrated in both the quantitative and qualitative results. Findings from the qualitative study shows that female respondents living in rural areas were less likely to report discrimination across all domains compared to females who live in urban areas. This finding reinforces the results from a previous study in Ethiopia, which observed that more young people in rural areas utilized sexual and reproductive health services compared to their colleagues in urban areas, and another study in Scotland, which documented more satisfaction with healthcare services among rural patients compared with urban and suburban residents [44, 45]. The findings underline the importance of considering specific contexts of rural and urban communities when addressing challenges associated with access to SRH services.

Consistent with an earlier study [46], the current study indicated that young people who are in school were more likely to experience discrimination when seeking SRH services compared with those who are out of school. This could be because healthcare providers are unconvinced of the level of maturity or responsibility of in-school youth compared to their out-of-school counterparts who may more likely be financially independent as they are often engaged in economic activities. This bias may lead to differential treatments.

Quantitative findings further demonstrated that the odds of discrimination were higher among respondents in urban areas, while the qualitative results provided more insight into how this discrimination manifests, particularly through negative provider attitudes. This is in keeping with findings from a prior study in Northern Nigeria, which identified poor provider attitude as one of the many challenges encountered by in-school young people when seeking SRH services [24]. The finding further affirms that discrimination is influenced by the convergence of different social identity markers [37]. This has consequences for SRH care of young people: it can deter them from seeking the care they need, contributing to a cycle of limited knowledge, unsafe practices, increased vulnerability to sexually transmitted infections, and unintended pregnancies. All of which result in poor SRH outcomes. This underscores the need for interventions that would foster supportive environments within health facilities and ensure that young people have more positive experiences when seeking SRH services.

### Implications for policy and practice

The findings of the study have crucial implications for improving SRH services for young people at PHCs. Efforts should be multi-pronged and addressing the intersecting factors. First, there is a need to develop and implement SRH policies that that promote inclusion of all young people irrespective of their age, gender, economic status, and schooling status. Second, healthcare providers should be trained to be more sensitive and inclusive in their interactions with diverse groups of young people, especially those with vulnerabilities. Third, efforts should be made to improve the overall reception at PHCs and promote a friendlier and welcoming environment. This could involve ongoing training programs for healthcare providers on youth-friendly health services. Moreover, owing to the higher likelihood of discrimination in urban areas, strategies to reduce discrimination in these settings should be prioritized. Finally, initiatives should be implemented to address the substantial gender and age-related disparities faced by female, in-school, and urban-dwelling young people. This may involve community engagement, awareness campaigns, and intersectionality-informed interventions. These measures can lead to inclusive and supportive facility environments for young people seeking SRH services at PHCs.

### Strengths and limitations of the study

This study was based on respondents’ self-reported behaviour, and this could have affected the accuracy of the responses leading to social desirability bias. However, the mixed methods approach ensured the triangulation of data from different sources and methods, and the intersectionality analysis enabled a more holistic understanding of the different intersecting social identities that influence the experience of discrimination by young people accessing SRH services. The findings from the qualitative interviews were shared with some of the participants for their validation.

## Conclusion

Our study identified the nature of discrimination that young people experience from SRH services, and the intersecting social identities that influence their experiences when seeking these services from PHCs. Health policies and programmes are needed to help reduce healthcare disparities and improve healthcare access for young people with various social identities. These policies should address age, gender, and education-based discrimination as well as other forms related to disability and socio-economic status. Intersectional perspectives should be prioritized in the design and implementation of capacity building interventions for health workers and community members. Through these measures, more inclusive and supportive environments can be promoted in primary health centres, leading to improved SRH outcomes for young people.

## Data Availability

The data set can be made available on request.
